# Machine Learning Modeling to Predict Atrial Fibrillation Detection in Embolic Stroke of Undetermined Source Patients

**DOI:** 10.3390/jpm14050534

**Published:** 2024-05-16

**Authors:** Chua Ming, Geraldine J. W. Lee, Yao Hao Teo, Yao Neng Teo, Emma M. S. Toh, Tony Y. W. Li, Chloe Yitian Guo, Jiayan Ding, Xinyan Zhou, Hock Luen Teoh, Swee-Chong Seow, Leonard L. L. Yeo, Ching-Hui Sia, Gregory Y. H. Lip, Mehul Motani, Benjamin YQ Tan

**Affiliations:** 1 Department of Medicine, Yong Loo Lin School of Medicine, National University of Singapore, Singapore 117597, Singapore; 2 Department of Statistics and Data Science, Faculty of Science, National University of Singapore, Singapore 117546, Singapore; 3 Department of Cardiology, National University Heart Centre, Singapore 119074, Singapore; 4 Division of Neurology, Department of Medicine, National University Hospital, Singapore 119074, Singapore; 5 Liverpool Centre for Cardiovascular Science at University of Liverpool, Liverpool John Moores University and Liverpool Heart & Chest Hospital, Liverpool L14 3PE, UK; 6 Danish Center for Health Services Research, Department of Clinical Medicine, Aalborg University, 9220 Aalborg, Denmark; 7 Department of Electrical and Computer Engineering, National University of Singapore, Singapore 117583, Singapore; 8 N.1 Institute for Health, National University of Singapore, Singapore 117456, Singapore; 9 Institute for Digital Medicine, Yong Loo Lin School of Medicine, National University of Singapore, Singapore 117456, Singapore; 10 Institute of Data Science, National University of Singapore, Singapore 117602, Singapore

**Keywords:** embolic stroke of undetermined source, atrial fibrillation, ischemic stroke, implantable loop recorder

## Abstract

Background: In patients with embolic stroke of undetermined source (ESUS), occult atrial fibrillation (AF) has been implicated as a key source of cardioembolism. However, only a minority acquire implantable cardiac loop recorders (ILRs) to detect occult paroxysmal AF, partly due to financial cost and procedural inconvenience. Without the initiation of appropriate anticoagulation, these patients are at risk of increased ischemic stroke recurrence. Hence, cost-effective and accurate methods of predicting AF in ESUS patients are highly sought after. Objective: We aimed to incorporate clinical and echocardiography data into machine learning (ML) algorithms for AF prediction on ILRs in ESUS. Methods: This was a single-center cohort study that included 157 consecutive patients diagnosed with ESUS from October 2014 to October 2017 who had ILR evaluation. We developed four ML models, with hyperparameters tuned, to predict AF detection on an ILR. Results: The median age of the cohort was 67 (IQR 59–74) years old and the median monitoring duration was 1051 (IQR 478–1287) days. Of the 157 patients, 32 (20.4%) had occult AF detected on the ILR. Support vector machine predicted for AF with a 95% confidence interval area under the receiver operating characteristic curve (AUC) of 0.736–0.737, multilayer perceptron with an AUC of 0.697–0.708, XGBoost with an AUC of 0.697–0.697, and random forest with an AUC of 0.663–0.674. ML feature importance found that age, HDL-C, and admitting heart rate were important non-echocardiography variables, while peak mitral A-wave velocity and left atrial volume were important echocardiography parameters aiding this prediction. Conclusion: Machine learning modeling incorporating clinical and echocardiographic variables predicted AF in ESUS patients with moderate accuracy.

## 1. Introduction

The annual burden of ischemic stroke (IS) has increased substantially in the past 20 years, with 12.2 million incident cases in 2019 [1]. Embolic stroke of undetermined source (ESUS) accounts for approximately 17% of acute IS cases and confers an increased stroke recurrence risk of 4–5% annually [2]. ESUS is defined as a non-lacunar brain infarct in the absence of extracranial or intracranial atherosclerosis, major-risk cardioembolic sources, and any other specific cause of stroke [3]. Proposed etiologies of ESUS include atrial cardiopathy, non-obstructive arterial atherosclerotic plaques, left ventricular (LV) systolic dysfunction, cardiac valvular disease, patent foramen ovale, and cancer [4,5].

In patients with ESUS, occult paroxysmal atrial fibrillation (AF) has been implicated as a key occult source of embolism. In the prospective ASSERT cohort study, 10% of patients with ESUS were found to have atrial tachyarrhythmias detected on an implantable cardiac loop recorder (ILR), with an increased hazard rate of 5.6 for clinical AF at 2.5 years [6]. An ILR allows for the prolonged recording of heart rhythms for up to three years, increasing the detection rate of paroxysmal cardiac arrhythmias such as AF [7]. In patients with ESUS, continuous heart rhythm monitoring using ILRs identifies AF in approximately 30% of these patients [8], and previous studies have shown that the presence of such paroxysmal AF episodes conferred an increased risk of stroke [6]. Despite current recommendations, only a minority of patients with ESUS receive prolonged cardiac monitoring owing to patient preferences, procedural inconvenience, and the significant cost of an ILR implantation [9,10]. This leads to a missed opportunity to institute ideal treatment with oral anticoagulation to mitigate the risk of a recurrent stroke. As such, cost-effective and accurate ways to risk stratify patients and predict for underlying occult AF in patients with ESUS are highly sought after.

Machine learning (ML) is a branch of artificial intelligence that utilizes data and algorithms to learn and make predictions. While there is established evidence regarding the association of echocardiographic parameters such as atrial and ventricular size, valvular heart disease, and left atrial thrombus with AF, ML using echocardiographic parameters has not been adequately applied to predict AF in patients with ESUS. The incorporation of echocardiographic parameters alongside clinical variables may enhance the predictive accuracy of ML models.

Hence, the aim of this study was to develop an ML prediction model to predict paroxysmal AF in patients with ESUS using a combination of clinical parameters, biomarkers, and echocardiographic parameters. We hypothesized that an ML model incorporating a combination of clinical and echocardiographic parameters will predict the occurrence of AF with moderate–high accuracy and provide insights on important variables aiding this prediction that may not be identified using traditional statistics.

## 2. Methods

### 2.1. Study Design

This study involved a retrospective cohort of consecutive patients with ESUS diagnosis from a stroke unit at a tertiary care hospital from October 2014 to October 2017. All 291 patients were offered an ILR, of which 157 proceeded with implantation and were included in the study and analyzed. Clinical and ILR data were collected from the institution’s electronic medical record and electronic ESUS database. The data collected comprised patient demographics, medical comorbidities, and laboratory and imaging results. Quantified data from echocardiography were recorded as categorical or numerical features and not run through the ML models as visual images. ESUS was defined according to the criteria outlined by the Cryptogenic Stroke/ESUS International Working Group as a non-lacunar brain infarction without the following: extracranial or intracranial atherosclerosis resulting in a luminal stenosis of 50% in the arteries supplying the area of infarction, major cardioembolic source, and other specific cause (e.g., vasculopathy, dissection, vasospasm, or thrombophilia) [3]. All the ILR data were extracted and evaluated by a trained electrophysiologist. This study is reported following the Strengthening the Reporting of Observational Studies in Epidemiology (STROBE) checklist [11]. Ethics approval was obtained from the National Healthcare Group Domain Specific Review Board (NHG DSRB Reference: 2021/00623). The study was conducted in accordance with the Declaration of Helsinki. Exemption for informed consent taking was given in view of the retrospective nature of the cohort and use of de-identified data.

### 2.2. Data Pre-Processing for Machine Learning Models

Descriptive statistics were used to compare characteristics between AF and non-AF groups, with Pearson’s Chi-squared test used for categorical variables and the Mann–Whitney U test for continuous variables. The dataset was first divided into 70% for the training set and 30% for the test set using stratified sampling from the Scikit-learn library [12]. IterativeImputer from the Scikit-learn library was used to impute missing values in numerical features, while missing values in categorical features were imputed using mode. Integer encoding was applied to ordinal variables, while one-hot encoding was applied to nominal variables to ensure compatibility with ML algorithms.

### 2.3. Overcoming an Imbalanced Dataset

Several resampling techniques were employed to balance the target distribution in the training set using the imbalanced-learn library in Python [13]. We implemented three broad resampling strategies, namely oversampling, undersampling, and a combination of both. We utilized two approaches to implement oversampling—random oversampling and the synthetic minority oversampling technique (SMOTE) [14]. Undersampling was implemented using random undersampling [15]. Furthermore, we proposed two combinations of oversampling and undersampling to balance the target distribution. Random oversampling and random undersampling were used jointly in the first combination while SMOTE and random undersampling were used jointly in the second combination.

### 2.4. Machine Learning Algorithms

Several ML algorithms, namely support vector machine (SVM), random forest, extreme gradient boosting (XGBoost), and multilayer perceptron (MLP), were implemented in Python version 3.9.12 with the aid of open-source packages from Scikit-learn version 1.2.0 and XGBoost version 1.7.2 [12]. Before resampling the training set, StandardScalar from the Scikit-learn library helped standardize numerical variables for SVM and MLP so that each variable had zero mean and unit variance.

SVM was implemented using Support Vector Classifier, random forest using Random Forest Classifier, XGBoost using XGBoost Classifier, and MLP using MLP Classifier, all of which were derived from the Scikit-learn library. Additionally, XGBoost leveraged the XGBoost library. Our MLP models contained one input layer, one hidden layer, and one output layer. The hyperparameters tuned using grid search for each ML model can be found in Supplementary Material: Table S1.

**Figure 1:**
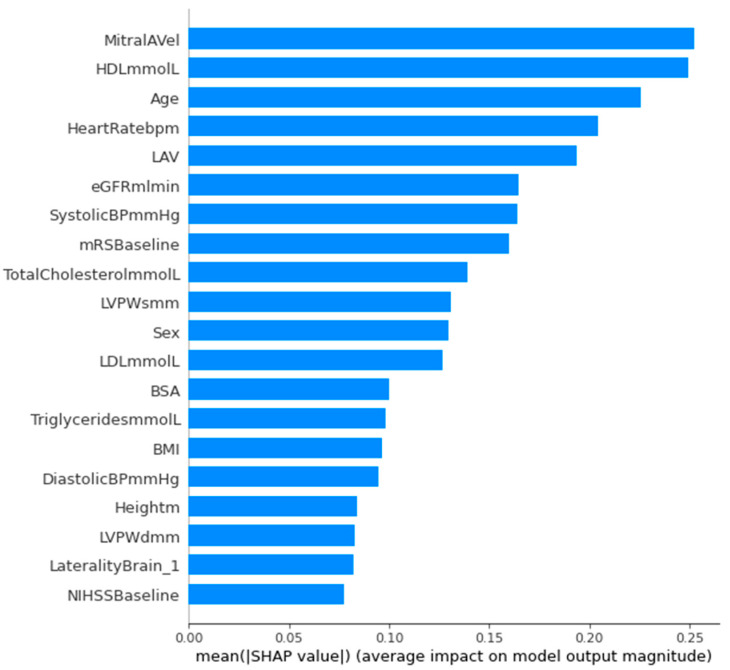
Mean absolute SHAP values of features in best-performing SVM model (top 20 features displayed). Abbreviations: SHAP, Shapley additive explanation; SVM, support vector machine. Abbreviations of all features can be found in Supplementary Materials: Table S2.

Feature selection was additionally performed on SVM with linear kernel, XGBoost, and random forest. Features were selected based on feature importance in the Scikit-learn library, which measured the individual contribution of each feature towards the performance of the respective classifier [16]. Thus, features with an absolute importance value greater than or equal to the specified threshold selected using grid search were retained in these models. Outside of feature selection models, feature importance values were also obtained for our best-performing random forest model (RF with random undersampling and hyperparameter tuning) without reducing the number of features (Supplementary Materials: Figure S1). Shapley additive explanation (SHAP) [17] was performed on our best-performing model, namely SVM with random oversampling and hyperparameter tuning (Figure 1), with mean absolute SHAP values obtained as a unified measure of feature importance.

### 2.5. Performance Metrics

For each ML model, the performance scores of 20 iterations were obtained, with each iteration using a different random state. In each iteration, a grid search with 5-fold cross-validation was utilized to select the best hyperparameter set for the model. The 95% confidence interval was calculated for each test performance metric by aggregating the results from 20 iterations. As the outcome was categorical, the summary performance estimates used included test AUC, accuracy, sensitivity, specificity, and F1 score.

## 3. Results

### 3.1. Baseline Characteristics

This study included 157 patients who had an ILR implantation after ESUS. The median age was 67 years (IQR 59–74), with 43 (27.4%) patients being female and 128 (81.5%) being of Chinese ethnicity. Patients were monitored for a median duration of 2.88 (IQR 1.31–3.52) years. There were 108 (68.8%) patients with hypertension, 96 (61.1%) with hyperlipidemia, 60 (38.2%) who were current or previous smokers, and 60 (38.2%) with diabetes mellitus. The median National Institutes of Health Stroke Scale (NIHSS) score was 4 (IQR 1.5–8.5). Of the 157 patients, 32 (20.4%) had AF detected on their ILR subsequently. Comparing the group with AF detected versus the group without, the median age and high-density lipoprotein cholesterol (HDL-C) were significantly higher, while the admitting heart rate was significantly lower (Table 1). Apart from the proportion of patients with mitral stenosis, there were no significant differences in echocardiography parameters between these two groups (Table 2).

### 3.2. Performance of ML Models

The following describes the best-performing model of each distinctive model type. The performance estimates of SVM with random oversampling and hyperparameter tuning comprised an AUC of 0.736–0.737, sensitivity of 0.600–0.600, and specificity of 0.816–0.816. XGBoost with random undersampling and hyperparameter tuning performed the best compared to other balancing techniques, with an AUC of 0.697–0.697, sensitivity of 0.900–0.900, and specificity of 0.395–0.395. Random forest with random undersampling and hyperparameter tuning had an AUC of 0.663–0.674, sensitivity of 0.650–0.700, and specificity of 0.492–0.516. The MLP model with random undersampling and hyperparameter tuning had an AUC of 0.697–0.708, sensitivity of 0.605–0.666, and specificity of 0.627–0.652 (Table 3).

### 3.3. Feature Importance

#### 3.3.1. Feature Importance Using Best-Performing Random Forest

The top five predictive features in AF detection on an ILR are heart rate, estimated glomerular filtration rate (eGFR), age, height, and HDL-C, before optimizing the number of features retained (Supplementary Materials: Figure S1).

#### 3.3.2. Feature Importance via SHAP in SVM

**Figure 2:**
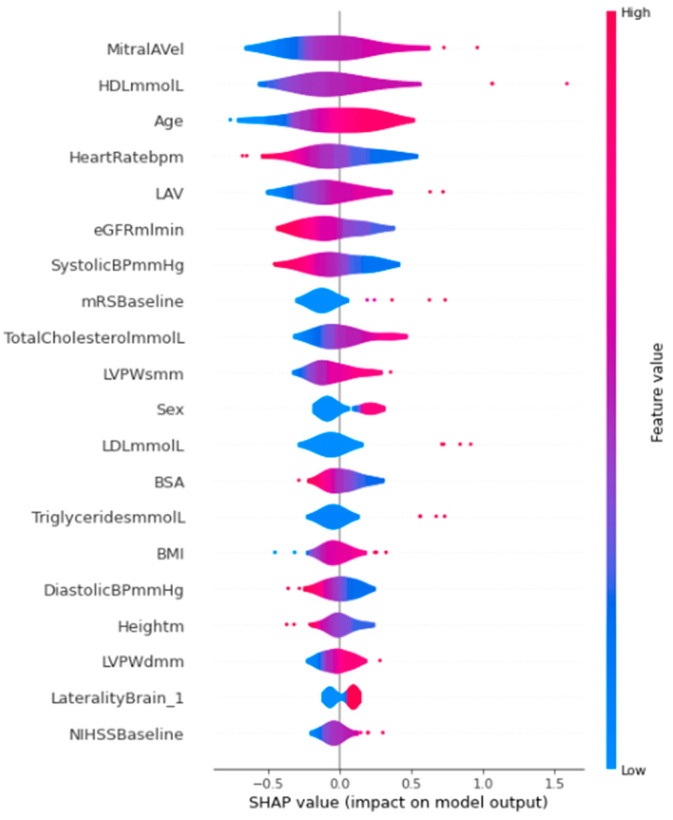
Violin plot of best-performing SVM model (top 20 features displayed). Abbreviations: SVM, support vector machine. Abbreviations of all features can be found in Supplementary Materials: Table S2.

The mean absolute SHAP values of features were obtained from our best performing model—SVM with random oversampling only and with hyperparameter tuning—of which the top 20 features are displayed in Figure 1. The five features with the highest mean absolute SHAP values were peak mitral A-wave velocity (MitralAVel), HDL-C, age, heart rate, and left atrial volume (LAV), three of which correspond with the five most important features in random forest (Figure 1 and Figure S1). Corresponding violin and beeswarm plots are visualized in Figure 2 and Figure S2, respectively. Force plots are visualized in Supplementary Materials: Figures S3 and S4.

### 3.4. Feature Selection Using SVM, Random Forest, and XGBoost

**Figure 3:**
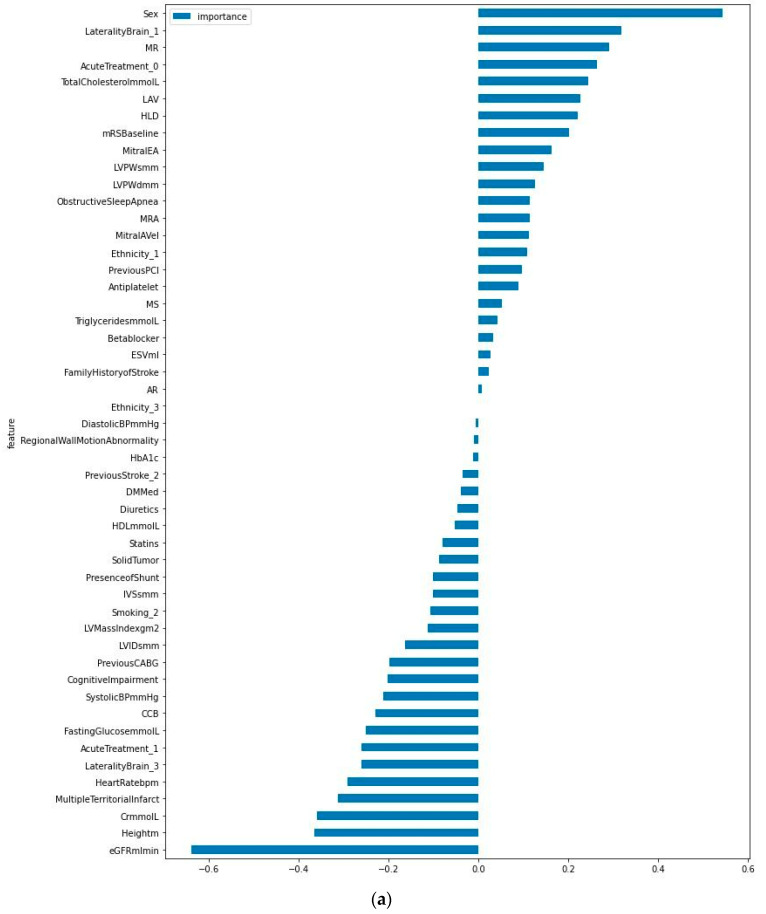

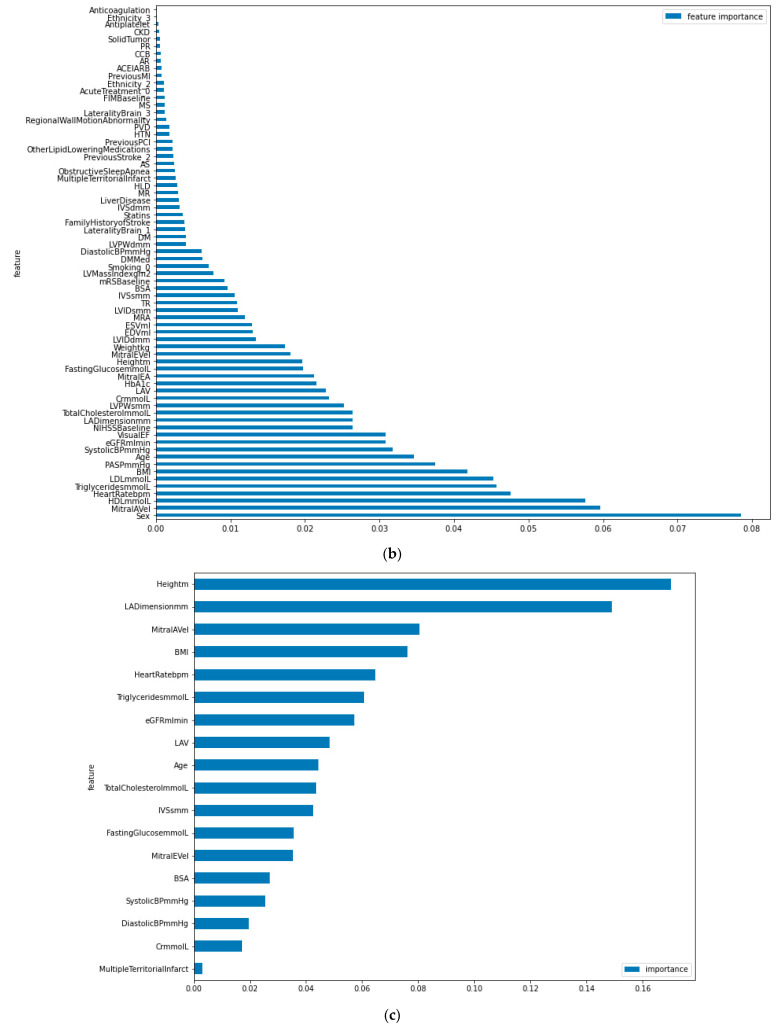
(**a**) Feature selection using SVM model with SMOTE; (**b**) feature selection using random forest with random undersampling; (**c**) feature selection using XGBoost with random undersampling. Abbreviations: SVM, support vector machine; SMOTE, synthetic minority oversampling technique; XGBoost, extreme gradient boosting. Abbreviations of all features can be found in Supplementary Materials: Table S2.

Feature selection using the SVM model resampled with SMOTE generated an AUC of 0.676–0.676, sensitivity of 0.200–0.200, and specificity of 0.868–0.868 and retained 50 features (Table 3). The top five most important features were eGFR, sex, height, creatinine levels, and laterality of stroke (left) (Figure 3a). Feature selection using random forest resampled with random undersampling generated an AUC of 0.529, sensitivity of 0.300, and specificity of 0.816 and retained 70 features (Table 3). The top five most important features were sex, peak mitral A-wave velocity, HDL-C, admitting heart rate, and triglyceride levels (Figure 3b). Feature selection using XGBoost resampled with random undersampling generated an AUC of 0.650, sensitivity of 0.700, and specificity of 0.526, while retaining 18 features (Table 3). The top five most important features were height, left atrial diameter, peak mitral A-wave velocity, BMI, and admitting heart rate (Figure 3c).

## 4. Discussion

In this study, we have presented a series of ML models to identify variables that may be important in the prediction of AF occurrence through ILR monitoring to aid decision-making for ILR implantation in ESUS. Our SVM model performed the best in this prediction (AUC = 0.736–0.737) when compared to XGBoost, random forest, and MLP.

To our knowledge, there has been little research specifically regarding the detection of pAF after ESUS using ML algorithms. Our study is one of the few to incorporate an extensive list of pre-reported echocardiogram results with clinical and biochemical data into the predictive modeling of AF on ILRs.

The C_2_HEST score was developed using a large French nationwide cohort to predict AF after ischemic stroke, comprising of coronary artery disease, chronic obstructive pulmonary disease, hypertension, advanced age, systolic heart failure, and thyroid disease [18]. Other scoring systems were previously developed using traditional statistics to predict AF specifically after cryptogenic stroke, namely the Brown ESUS-AF [19], HAVOC [20], AS5F [21], and AF-ESUS scores [22]. The Brown ESUS-AF score was developed using multivariable logistic regression, comprising age and left atrial enlargement, which predicted AF using cardiac monitoring in ESUS [19]. The HAVOC score stratified patients into low-, medium-, and high-risk groups and evaluated the probability of AF detection after ESUS in each group using seven clinical variables, namely age, obesity, congestive heart failure, hypertension, coronary artery disease, peripheral vascular disease, and valve disease [20]. The AS5F score comprised age and stroke severity [21]. The AF-ESUS score assigned positive weights for age, hypertension, left atrial diameter > 40 mm, and supraventricular extrasystole and negative weights for left ventricular hypertrophy, left ventricular ejection fraction < 35%, subcortical infarct, and non-stenotic carotid plaques [22].

Comparing random forest’s feature importance to the feature importance via mean absolute SHAP values using SVM, among the ten most important features in each of the two models, six were identical (admitting heart rate, eGFR, age, HDL-C, peak mitral A-wave velocity, and systolic blood pressure). Among the top five most important features in each of the two models, three were identical (age, HDL-C, and admitting heart rate) (Figure 1 and Figure S1). SHAP values provide an interpretable approximation of the original model with a unique additive measure that adheres to three desirable properties and defines simplified inputs using conditional expectations [17]. Amongst all the aforementioned clinical scores and features of importance in our ML models, age was identified as an important predictor of pAF detection in ESUS. This corroborates the results of past studies that found age to be a predictor of AF in ESUS [23] and age above 60 years old to be a robust indicator of occult AF after cryptogenic stroke [24]. The mechanism could be attributed to age-related atrial myocardial electrical and structural remodeling [25]. Thus, this reinstates the usefulness of age to stratify patients who require extended cardiac monitoring in ESUS. Mitral A-wave velocity is an echocardiography parameter that assesses blood flow through the mitral valve due to atrial contraction. While it is poorly understood whether transmitral inflow waves and filling can be used to predict AF, studies have reported that patients with progression to permanent AF had lower peak A velocity than those without progression [26]. In our study, peak mitral A-wave velocity was identified as an important variable in feature selection using random forest and XGBoost (Figure 3b,c). However, no association was found on traditional descriptive statistics.

Left atrial volume was identified as important in our ML feature importance assessment, which concurs with a past study that found left atrial volume to be significantly higher in the group with AF detected via ILR in unexplained stroke [27]. Similarly, left atrial enlargement and left atrial diameter are also variables included in the Brown ESUS-AF score and AF-ESUS score, respectively.

Estimated glomerular filtration rate (eGFR) was the second most important feature in our best-performing random forest model and the most important feature in SVM with feature selection (Supplementary Materials: Figures S1 and S3). An explanation is that AF is a prothrombotic state that causes microthrombi of the renal vasculature. This reduces renal perfusion and results in renal ischemia, causing kidney impairment reflected as reduced eGFR [28]. The converse process may be plausible as well. Initial renin–angiotensin–aldosterone system (RAAS) activation in patients with renal impairment may itself be arrhythmogenic, where oxidative stress mediates changes in cellular ion channels [29], resulting in paroxysmal AF. Furthermore, the RAAS has been found to be closely connected to AF development through inflammation and structural and electrical cardiac remodeling [30]. Sex was identified as the most important and second most important feature in the random forest and SVM models, respectively. It is possible that the attributable proportions of etiologies of ESUS may differ between genders. In males, the size, composition, and morphology of carotid atherosclerotic plaques were found to be more pronounced than in females [31]; thus, ESUS etiology in males may be more greatly attributed to non-stenotic atherosclerotic plaques than paroxysmal AF. In the AF-ESUS score, variables with negative weights assigned were more predictive of an absence of new AF detection. Similarly, our ML models were able to identify an ordered list of features that are predictive of absence of AF detection on an ILR, such as eGFR, height, and systolic blood pressure (Figure 2 and Figure 3a).

An extensive 48-country survey of stroke units found prolonged cardiac monitoring not to be a routine workup for cryptogenic stroke even in high-income nations [10]. With ESUS patients having a notable risk of stroke recurrence of 4–5% yearly [22], optimizing resource allocation for patients who require prolonged cardiac monitoring after ESUS remains important to reduce the high costs associated with implementing prolonged cardiac monitoring. Our study provides an ML approach to aid decision-making for prolonged cardiac monitoring in ESUS patients and provides future studies with a supplemental list of variables for evaluation.

## 5. Strengths and Limitations

Machine learning models have been used to predict outcomes of stroke. However, reporting standards have been suboptimal, such as the exclusion of hyperparameter selection reporting, lack of clear reporting regarding the handling of imbalanced datasets, and the absence of feature selection [32]. In our study, we have presented four distinct ML models with data imbalance addressed and hyperparameters tuned and with feature selection for selected models. Multiple resampling techniques were evaluated to handle the imbalanced target class, including random oversampling, random undersampling, SMOTE, and a combination of these, which facilitated the development of the best performing model for each distinctive ML type. The directional relationship of variables with outcomes should be observed using our violin plots of SHAP values generated with our best performing SVM model and the SVM feature selection plot (Figure 2 and Figure 3a) instead of our random forest feature importance plot as the latter was unable to discern direction of outcome prediction. Thus, absolute predictive ability should not be confused with directional predictability. Internal validation was performed through performance evaluation on unseen validation (test) data. Generalizability should be further evaluated in the future. The performance of our MLP model should be interpreted with discretion as it is a neural network algorithm limited by a relatively small patient cohort. In our study, many features were used to predict the presence of AF on ILRs, compared to several used in existing clinical scores. In the pre-processing stage, imputation of missing values of categorical features may be explored using multiple imputations to compare with the current imputation by mode. Future studies should further explore discordances between important features identified via descriptive statistics and traditional logistic regression compared to machine learning modalities in larger international ESUS datasets.

## 6. Conclusions

Machine learning modeling incorporating clinical and echocardiographic variables predicted AF in ESUS patients with moderate accuracy.

## Figures and Tables

**Table 1:** Characteristics of patients with ILR implantation after ESUS, with comparison between AF detected on ILR and AF not detected on ILR.

Variable	Total (*N* = 157)	AF Not Detected on ILR (n = 125)	AF Detected on ILR (n = 32)	*p*-Value
Age (years, median [IQR])	67.00 [59.00, 74.00]	65.00 [57.00, 72.00]	73.50 [67.00, 77.25]	<0.001
Female sex (n, %)	43 (27.4)	28 (22.4)	15 (46.9)	0.011
Duration of ILR monitoring (days, median [IQR])	1051.00 [478.00, 1287.00]	1024.00 [387.00, 1287.00]	1093.00 [664.25, 1283.50]	0.33
Ethnicity (n, %)				0.442
Chinese	128 (81.5)	100 (80.0)	28 (87.5)	
Malay	12 (7.6)	9 (7.2)	3 (9.4)	
Indian	12 (7.6)	11 (8.8)	1 (3.1)	
Others	5 (3.2)	5 (4.0)	0 (0.0)	
Comorbidities (n, %)				
Smoking status				0.103
Never	97 (61.8)	72 (57.6)	25 (78.1)	
Previous smoker	52 (33.1)	46 (36.8)	6 (18.8)	
Current smoker	8 (5.1)	7 (5.6)	1 (3.1)	
Hypertension	108 (68.8)	83 (66.4)	25 (78.1)	0.288
Hyperlipidemia	96 (61.1)	76 (60.8)	20 (62.5)	1
Diabetes mellitus status				0.604
No DM	97 (61.8)	75 (60.0)	22 (68.8)	
DM on OHGA	56 (35.7)	47 (37.6)	9 (28.1)	
DM on insulin	4 (2.5)	3 (2.4)	1 (3.1)	
Stroke Parameters				
Acute treatment (n/total, %)				0.4
No tPA or EVT	111/152 (73.0)	87/122 (71.3)	24/30 (80.0)	
tPA	23/152 (15.1)	21/122 (17.2)	2/30 (6.7)	
EVT	10/152 (6.6)	7/122 (5.7)	3/30 (10.0)	
Both tPA and EVT	8/152 (5.3)	7/122 (5.7)	1/30 (3.3)	
Baseline mRS (n/total, %)				0.084
0	91/114 (79.8)	75/92 (81.5)	16/22 (72.7)	
1	13/114 (11.4)	12/92 (13.0)	1/22 (4.5)	
2	3/114 (2.6)	2/92 (2.2)	1/22 (4.5)	
3	5/114 (4.4)	2/92 (2.2)	3/22 (13.6)	
4	2/114 (1.8)	1/92 (1.1)	1/22 (4.5)	
Admitting NIHSS (median [IQR])	4.00 [1.50, 8.50]	4.00 [2.00, 7.75]	3.00 [1.00, 14.00]	0.78
Admitting systolic BP (mmHg, median [IQR])	152.00 [132.00, 170.00]	150.00 [132.25, 168.50]	162.00 [129.50, 175.00]	0.49
Admitting diastolic BP (mmHg, median [IQR])	82.00 [72.00, 92.00]	82.50 [72.00, 92.75]	82.00 [72.50, 86.00]	0.416
Admitting heart rate (beats per minute, median [IQR])	78.00 [68.00, 88.00]	80.00 [71.50, 89.00]	68.00 [63.00, 82.00]	0.003
Biochemical Parameters				
Total cholesterol (mmol/L, median [IQR])	4.56 [3.80, 5.34]	4.53 [3.78, 5.32]	4.84 [4.22, 5.81]	0.085
LDL (mmol/L, median [IQR])	2.88 [2.03, 3.54]	2.77 [1.94, 3.49]	3.02 [2.28, 4.24]	0.079
HDL (mmol/L, median [IQR])	1.15 [1.00, 1.35]	1.13 [1.00, 1.30]	1.34 [1.06, 1.67]	0.004
HbA1c (%, median [IQR])	6.05 [5.60, 6.93]	6.20 [5.70, 7.07]	5.90 [5.53, 6.50]	0.207
Fasting glucose (mmol/L, median [IQR])	5.70 [5.20, 6.50]	5.70 [5.30, 6.70]	5.45 [5.20, 5.97]	0.217
Serum creatinine (µmol/L, median [IQR])	77.00 [61.50, 93.50]	76.00 [62.25, 90.00]	81.00 [58.00, 100.00]	0.419
eGFR (mL/min/1.73 m^2^, median [IQR])	89.00 [69.25, 99.00]	91.00 [75.25, 100.00]	76.00 [57.50, 91.00]	0.012
Others				
Previous myocardial infarction (n, %)	21 (13.4)	16 (12.8)	5 (15.6)	0.898
Previous stroke (n, %)				0.682
None	98 (62.4)	76 (60.8)	22 (68.8)	
TIA	45 (28.7)	37 (29.6)	8 (25.0)	
Stroke	14 (8.9)	12 (9.6)	2 (6.2)	
Heart failure	7 (4.5)	5 (4.0)	2 (6.2)	0.944
Antiplatelet	141 (89.8)	113 (90.4)	28 (87.5)	0.876
Anticoagulation	12 (7.6)	11 (8.8)	1 (3.1)	0.481
Body mass index (kg/m^2^, median [IQR])	24.09 [21.53, 27.22]	24.09 [21.69, 27.14]	23.68 [21.42, 28.89]	0.893
Body surface area (median [IQR])	1.71 [1.57, 1.83]	1.72 [1.57, 1.83]	1.64 [1.54, 1.80]	0.238

Abbreviations: n, number; Total, total number of non-missing values; *p*, *p*-value of Pearson’s Chi-squared test for categorical variables and Mann–Whitney U test for continuous variables; IQR, interquartile range; ILR, implantable loop recorder; ESUS, embolic stroke of undetermined source; DM, diabetes mellitus; OHGA, oral hypoglycemic agent; tPA, tissue plasminogen activator; EVT, endovascular therapy; mRS, modified Rankin Scale; NIHSS, National Institutes of Health Stroke Scale; BP, blood pressure; LDL, low-density lipoprotein; HDL, high-density lipoprotein; HbA1c, hemoglobin A1c; eGFR, estimated glomerular filtration rate; TIA, transient ischemic attack.

**Table 2:** Characteristics of patients with ILR implantation after ESUS, with comparison between AF detected on ILR and AF not detected on ILR (echocardiography parameters).

Echocardiography Variable	Total (*N* = 157)	AF Not Detected on ILR (n = 125)	AF Detected on ILR (n = 32)	*p*-Value
Peak mitral E-wave velocity (cm/s, median [IQR])	63.40 [53.22, 80.40]	62.30 [54.00, 79.90]	69.30 [52.15, 81.38]	0.624
Peak mitral A-wave velocity (cm/s, median [IQR])	79.00 [63.33, 92.27]	78.10 [62.97, 88.70]	89.50 [66.75, 106.00]	0.105
Mitral E/A ratio (median [IQR])	0.86 [0.67, 1.04]	0.88 [0.67, 1.06]	0.74 [0.66, 0.89]	0.277
PASP (mmHg, median [IQR])	29.00 [26.00, 34.00]	29.00 [26.00, 34.00]	29.50 [26.50, 34.25]	0.842
LVIDd (mm, median [IQR])	47.00 [43.00, 51.00]	47.00 [43.00, 51.00]	48.00 [42.75, 51.00]	0.866
LVIDs (mm, median [IQR])	30.00 [26.00, 33.00]	30.00 [26.00, 33.00]	29.50 [25.75, 33.00]	0.867
EDV (ml, median [IQR])	102.00 [79.00, 124.00]	99.50 [79.00, 124.00]	108.00 [82.00, 124.00]	0.719
ESV (ml, median [IQR])	32.00 [25.00, 44.00]	32.00 [25.00, 44.00]	33.35 [24.25, 44.00]	0.965
VisualEF (%, median [IQR])	63.00 [60.00, 65.00]	62.00 [60.00, 65.00]	65.00 [57.75, 65.00]	0.734
IVSd (mm, median [IQR])	10.00 [9.00, 12.00]	10.00 [9.00, 12.00]	10.00 [9.00, 12.00]	0.931
IVSs (mm, median [IQR])	14.00 [12.00, 16.00]	14.00 [12.00, 16.00]	14.00 [12.75, 16.50]	0.926
LVPWd (mm, median [IQR])	10.00 [9.00, 11.00]	10.00 [8.50, 11.00]	10.00 [9.00, 11.00]	0.483
LVPWs (mm, median [IQR])	15.00 [13.00, 16.00]	15.00 [13.00, 16.00]	14.00 [13.00, 16.00]	0.83
Left atrial diameter (mm, median [IQR])	37.00 [33.00, 41.00]	37.00 [33.00, 41.00]	39.00 [35.75, 42.25]	0.077
Left atrial volume (ml, median [IQR])	45.00 [36.32, 56.30]	44.90 [34.20, 55.00]	50.81 [38.62, 61.61]	0.088
LV mass index (g/m^2^, median [IQR])	90.00 [77.00, 109.00]	89.00 [76.00, 106.00]	95.00 [79.50, 110.25]	0.701
MS (n, %)				0.017
None	154 (98.1)	124 (99.2)	30 (93.8)	
Mild	2 (1.3)	0 (0.0)	2 (6.2)	
Severe	1 (0.6)	1 (0.8)	0 (0.0)	

Abbreviations: n, total number of non-missing values; *p*, *p*-value of Pearson’s Chi-squared test for categorical variables and Mann–Whitney U test for continuous variables; IQR, interquartile range. Abbreviations of all features can be found in Supplementary Materials: Table S2.

**Table 3:** Performance scores of models with and without feature selection, with hyperparameters tuned.

ML Models without Feature Selection							
**Model**	**Balancing Strategy**	**Accuracy**	**Sensitivity**	**Specificity**	**F1 Score**	**AUC**	
XGBoost	Random undersampling	0.5–0.5	0.9–0.9	0.3947–0.3947	0.4286–0.4286	0.6974–0.6974	
Random Forest	Random undersampling	0.5290–0.5502	0.6500–0.7000	0.4915–0.5164	0.3671–0.3911	0.6629–0.6737	
SVM	Random oversampling	0.7708–0.7708	0.6–0.6	0.8158–0.8158	0.5217–0.5217	0.7362–0.7370	
SVM	Random undersampling	0.625–0.625	0.7–0.7	0.6053–0.6053	0.4375–0.4375	0.4863–0.6935	
SVM	Combined random oversampling and undersampling	0.7083–0.7083	0.6–0.6	0.7368–0.7368	0.4615–0.4615	0.7290–0.7290	
SVM	Combined SMOTE and random undersampling	0.7917–0.7917	0.4–0.4	0.8947–0.8947	0.4444–0.4444	0.6815–0.6818	
MLP	SMOTE	0.7271–0.7687	0.3781–0.4520	0.8136–0.8574	0.3714–0.4458	0.6497–0.6756	
MLP	Random oversampling	0.7049–0.7264	0.3928–0.4373	0.7789–0.8106	0.3638–0.3920	0.6559–0.6839	
MLP	Random undersampling	0.6262–0.6509	0.6045–0.6655	0.6271–0.6518	0.4041–0.4408	0.6973–0.7079	
ML Models with Feature Selection							
Model	Balancing Strategy	Number of Features Retained	Accuracy	Sensitivity	Specificity	F1 Score	AUC
SVM	SMOTE	50	0.7292–0.7292	0.2000–0.2000	0.8684–0.8684	0.2353–0.2353	0.6763–0.6763
Random Forest	Random undersampling	70	0.7083	0.3000	0.8158	0.3000	0.5289
XGBoost	Random undersampling	18	0.5625	0.7000	0.5263	0.4000	0.6500

Abbreviations: SVM, support vector machine; XGBoost, extreme gradient boosting; MLP, multilayer perceptron; AUC, area under the receiver operating characteristic curve; SMOTE, synthetic minority oversampling technique.

## Data Availability

Raw data are not publicly available due to ethical considerations. Further enquiries can be directed to the corresponding author.

## Supplementary Materials

The following supporting information can be downloaded at: https://www.mdpi.com/article/10.3390/jpm14050534/s1. Figure S1: Feature Importance of Best-performing Random Forest Model; Figure S2: Beeswarm Plot of Best-performing SVM Model (top 20 features displayed); Figure S3: SHAP Global Importance/Explanations: Force Plot with Best-performing SVM Model; Figure S4: SHAP Local Importance/Explanations: Force Plot with Best-performing SVM Model for single patient (index patient); Table S1: Set of Hyperparameters Tuned using Grid Search in Machine Learning Models; Table S2: List of Features included in ML Models and their Abbreviations.
