# Supplementary material for: Machine Learning Modeling to Predict Atrial Fibrillation Detection in Embolic Stroke of Undetermined Source Patients
Source: J Pers Med. 2024 May 16;14(5):534. doi: 10.3390/jpm14050534 (PMC11122555; doi:10.3390/jpm14050534)
Supplement: Supplementary file 1 [file jpm-14-00534-s001.zip › jpm-2983416-supplementary.pdf]

# Supplementary Material

Figure S1: Feature Importance of Best-performing Random Forest Model

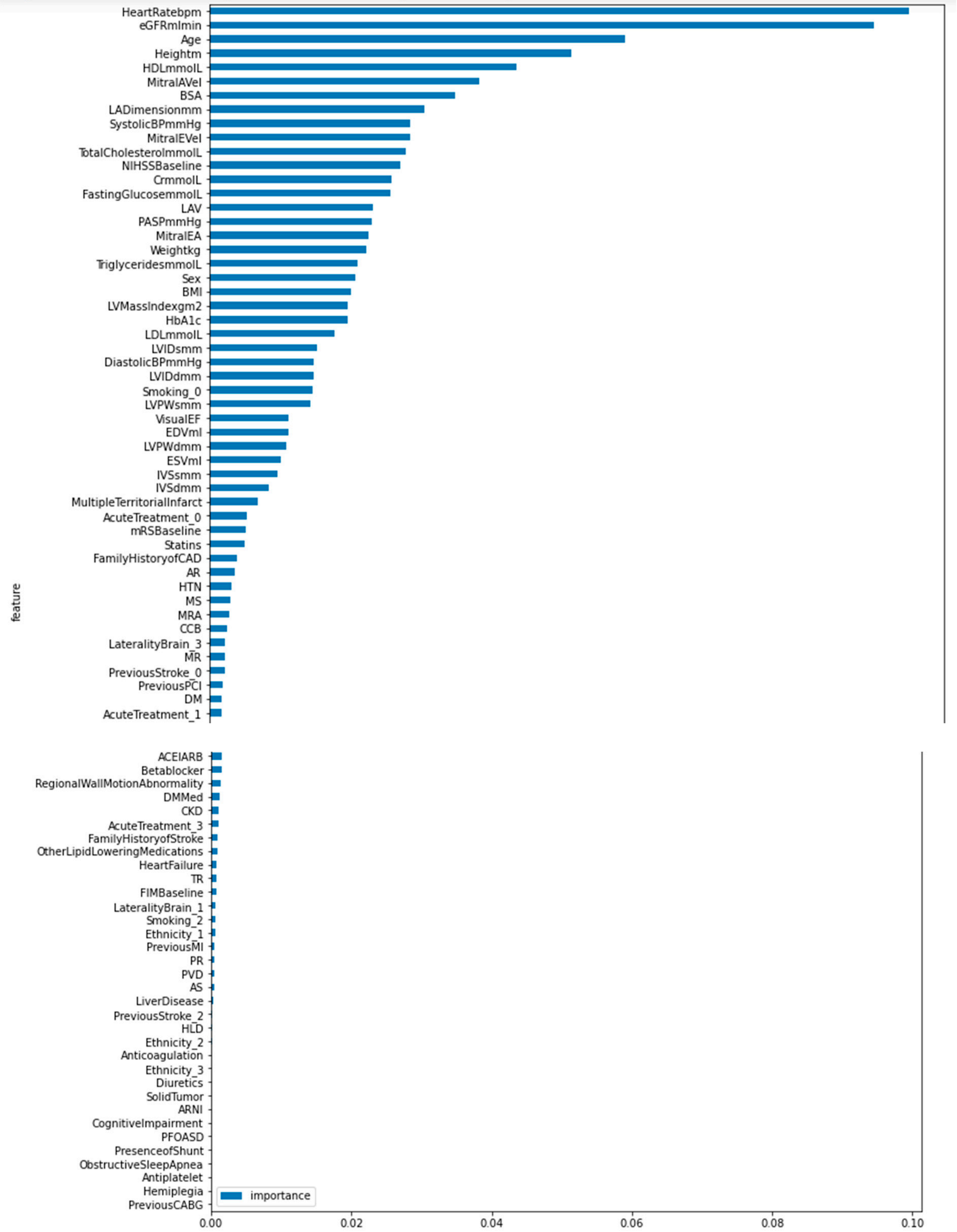


Abbreviations: abbreviations of all features are found in Supplementary Table 5.

Figure S2: Beeswarm Plot of Best-performing SVM Model (top 20 features displayed)

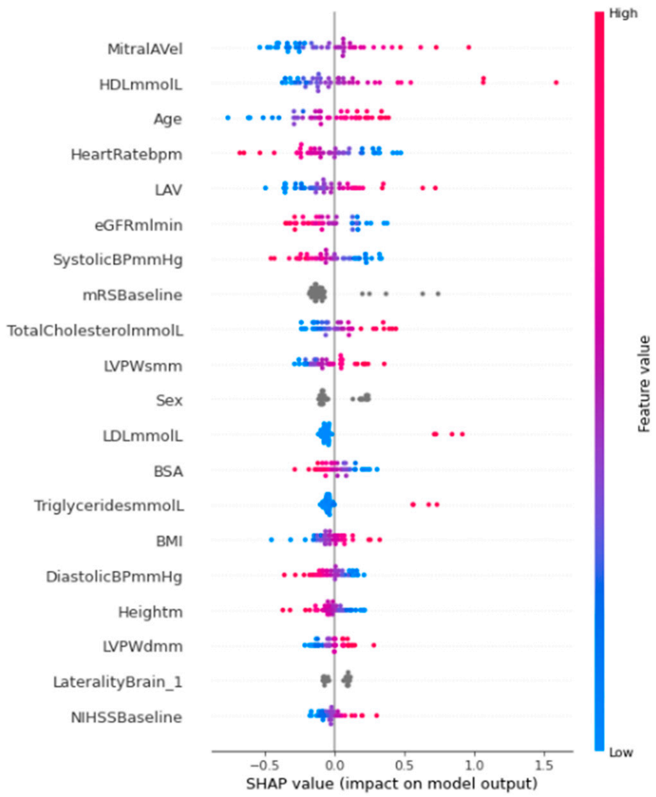


Abbreviations: SVM support vector machine. Abbreviations of all features are found in Supplementary Table 4.

Figure S3: SHAP Global Importance/Explanations: Force Plot with Best-performing SVM Model

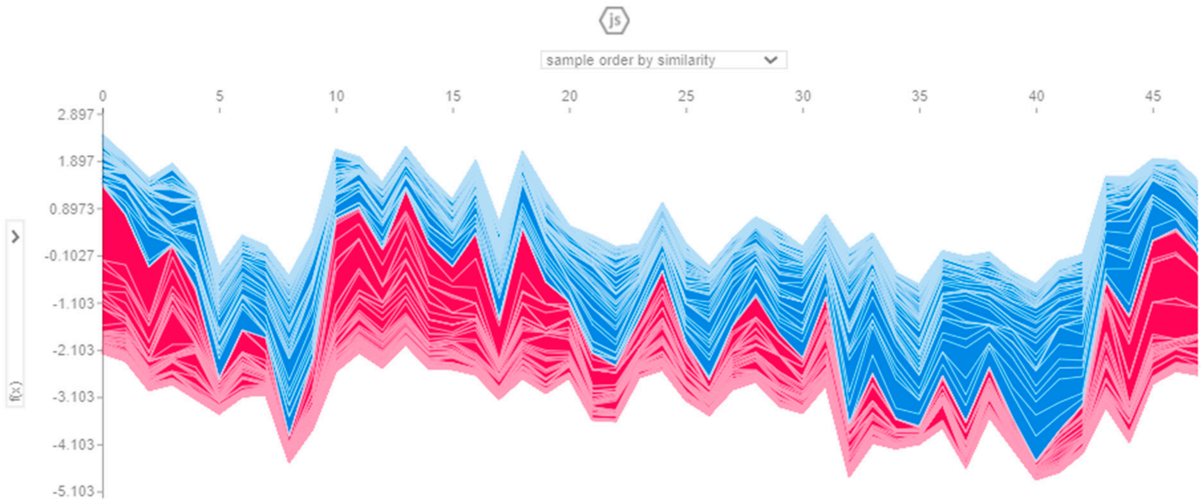


Abbreviations: SHapley Additive exPlanations, SVM support vector machine.

Figure S4: SHAP Local Importance/Explanations: Force Plot with Best-performing SVM Model for single patient (index patient)

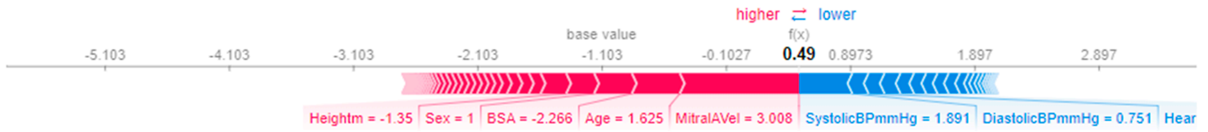


Abbreviations: SHapley Additive exPlanations, SVM support vector machine. Abbreviations of all features are found in Supplementary Table 4.

Table S1: Set of Hyperparameters Tuned using Grid Search in Machine Learning Models

| Model | Hyperparameters Tuned using Grid Search |
|---|---|
| SVM | misclassification penalty parameter C, gamma, and kernel function. |
| Random Forest | number of trees in the random forest, maximum depth of a tree, minimum number of samples required for each split, minimum number of samples required for each leaf node, and whether bootstrap samples are used when building trees. |
| XGBoost | learning rate, L1 regularization parameter, minimum loss reduction required for each leaf node, maximum tree depth for the base classifiers, minimum weight needed to create a new node, and the number of boosting iterations. |
| MLP | number of neurons in the hidden layer, activation function used for the hidden layer, the L2 regularization parameter, learning rate, and maximum number of iterations. |

Abbreviations: SVM support vector machine, XGBoost eXtreme Gradient Boosting, MLP multilayer perceptron

Table S2: List of Features included in ML Models and their Abbreviations:

| **Clinical parameters and biomarkers** | | **Echocardiography parameters** | |
|---|---|---|---|
| **Feature** | **Abbreviation used** | **Feature** | **Abbreviation used** |
| Age | Age | Cardiac shunt | PresenceofShunt |
| Sex | Sex | Peak mitral E-wave velocity | MitralEVel |
| Body surface area | BSA | Peak mitral A-wave velocity | MitralAVel |
| Ethnicity | Ethnicity | Mitral E/A ratio | MitralEA |
| Height | Heightm | PASP | PASPmmHg |
| Weight | Weightkg | LVID at end diastole | LVIDdmm |
| Systolic blood pressure | SystolicBPmmHg | LVID at end systole | LVIDsmm |
| Diastolic blood pressure | DiastolicBPmmHg | End-diastolic volume | EDVml |
| Admitting heart rate | HeartRatebpm | End-systolic volume | ESVml |
| Family history of coronary artery disease | FamilyHistoryofCAD | Ejection fraction | VisualEF |
| Family history of stroke | FamilyHistoryofStroke | Interventricular septal thickness in diastole | IVSdmm |
| Smoking | Smoking | Interventricular septal thickness in systole | IVSsmm |
| Hypertension | HTN | LVPWd | LVPWdmm |
| Hyperlipidemia | HLD | LVPWs | LVPWsmm |
| Diabetes mellitus status | DM | Left atrial diameter | LADimensionmm |
| Previous myocardial infarction | PreviousMI | Left atrial volume | LAV |
| Previous percutaneous coronary intervention | PreviousPCI | Left ventricular mass index | LVMassIndexgm2 |
| Previous coronary artery bypass graft surgery | PreviousCABG | PFO or ASD | PFOASD |
| Previous stroke | PreviousStroke | Mitral regurgitation | MR |
| Heart failure | HeartFailure | Mitral stenosis | MS |
| Peripheral vascular disease | PVD | Aortic regurgitation | AR |
| Cognitive impairment | CognitiveImpairment | Aortic stenosis | AS |
| Presence of liver disease | LiverDisease | Tricuspid regurgitation | TR |
| Hemiplegia | Hemiplegia | Pulmonary regurgitation | PR |
| Obstructive sleep apnea | ObstructiveSleepApnea | Regional wall motion abnormality | RegionalWallMotion-Abnormality |
| Chronic kidney disease | CKD | | |

| | | | |
|---|---|---|---|
| Solid tumor present | SolidTumor | | |
| **Medications** | | | |
| Beta blocker use | Betablocker | | |
| ACEI or ARB use | ACEIARB | | |
| Angiotensin Receptor Neprilysin inhibitor | ARNI | | |
| Mineralocorticoid receptor antagonist | MRA | | |
| Diabetic medication use | DMMed | | |
| Diuretics | Diuretics | | |
| Calcium channel blocker | CCB | | |
| Statin use | Statins | | |
| On non-statin lipid-lowering medications | OtherLipidLoweringMedications | | |
| Antiplatelet | Antiplatelet | | |
| Anticoagulation | Anticoagulation | | |
| **Stroke parameters** | | | |
| Laterality of stroke | LateralityBrain | | |
| Multiple territorial infarct | MultipleTerritorialInfarct | | |
| Acute treatment for stroke | AcuteTreatment | | |
| Functional Independent Measure at baseline | FIMBaseline | | |
| Modified Rankin Scale at baseline | mRSBaseline | | |
| NIHSS at baseline | NIHSSBaseline | | |
| ASPECTS at baseline | ASPECTSBaseline | | |
| Total cholesterol | TotalCholesterolmmolL | | |
| LDL-C | LDLmmolL | | |
| HDL-C | HDLmmolL | | |
| Triglycerides | TriglyceridesmmolL | | |
| HbA1c | HbA1c | | |
| Fasting glucose | FastingGlucosemmolL | | |
| Creatinine | CrmmolL | | |
| eGFR | eGFRmlmin | | |

Abbreviations: ACEI angiotensin-converting enzyme inhibitor, ARB angiotensin receptor blocker, ASPECTS Alberta Stroke Program Early CT Score, NIHSS National Institutes of Health Stroke Scale, LDL-C low-density lipoprotein cholesterol, HDL-C high-density lipoprotein cholesterol, HbA1c hemoglobin A1c, eGFR estimated glomerular filtration rate, PASP pulmonary arterial systolic pressure, LVID left ventricular internal diameter, LVPWd left ventricular posterior wall thickness end-diastole, LVPWs left ventricular posterior wall thickness end-systole, PFO patent foramen ovale, ASD atrial septal defect
